# The Indirect ELISA *Trypanosoma evansi* in Equids: Optimisation and Application to a Serological Survey including Racing Horses, in Thailand

**DOI:** 10.1155/2019/2964639

**Published:** 2019-12-05

**Authors:** Margot Camoin, Arthur Kocher, Piangjai Chalermwong, Sarawut Yangtarra, Ketsarin Kamyingkird, Sathaporn Jittapalapong, Marc Desquesnes

**Affiliations:** ^1^Centre de Coopération Internationale en Recherche Agronomique pour le Développement (CIRAD), UMR InterTryp, Kasetsart University, Chatuchak, 10900 Bangkok, Thailand; ^2^UMR InterTryp, Université de Montpellier, CIRAD, IRD, 34398 Montpellier, France; ^3^Faculty of Veterinary Medicine, Kasetsart University, Chatuchak, 10900 Bangkok, Thailand; ^4^Faculty of Veterinary Technology, Kasetsart University, Chatuchak, 10900 Bangkok, Thailand

## Abstract

Surra, caused by *Trypanosoma evansi,* is a widely distributed animal trypanosomosis; it affects both domestic and wild mammals with high economic impact. Clinical picture is moderate in bovines but severe in equids. Surra is also an important constraint for international animal trade and movements. Despite its impact, surra remains poorly diagnosed because of low sensitivity tests. To improve epidemiological knowledge of the disease and to secure international movement, efficient diagnosis tools are required. Here, we optimized and applied to equids the OIE-recommended indirect ELISA *T. evansi* that was validated in other species. Based on 96 positive and 1,382 negative horse reference samples from Thailand, a TG-ROC analysis was conducted to define the cutoff value. ELISA's sensitivity and specificity were estimated at 97.5% and 100%, respectively, qualifying the test to provide a reliable immune status of equids. The test was then applied on 1,961 horse samples from 18 Thai Provinces; the only scarce positives suggested that horses do not constitute a reservoir of *T. evansi* in Thailand. All samples from racing horses were negative. Conversely, two outbreaks of surra reported to our laboratory, originating from a bovine reservoir, exhibited high morbidity and lethality rates in horses. Finally, posttreatment follow-ups of infected animals allowed us to provide outbreak management guidelines.

## 1. Introduction

Most parasitic diseases have a higher impact on animal health in tropical compared to temperate areas; it is mainly due to a lack of appropriate control measures or climatic conditions, and it is especially true for improved imported breeds [1–3]. Amongst them, trypanosomosis hinders livestock farming development in many tropical countries. Indeed, *Trypanosoma evansi*, the causative agent of surra, is the animal trypanosome that has the widest geographical repartition [4]. The worldwide success of *T. evansi*, compared to other African trypanosomes whose geographical distribution remains within the tsetse belt, is due to its adaption to mechanical transmission by biting insects, such as tabanids and stomoxes [5]. *Trypanosoma evansi* is endemic in America, Africa, and Asia, and its implantation in continental Europe is also to be feared after sporadic imported cases were observed in France and Spain [6, 7]. Therefore, high sensitivity methods for the detection of Surra infection appear to be a prerequisite to animal international trade and other animal movements.

Surra affects a wide host range: both domestic and wild species can exhibit polymorphic clinical signs. Equids, camelids, and dogs undergo acute forms most often leading to death. Clinical signs in horses include fever, weight loss with no depression of appetite, bilateral epiphora, anaemia, and dependent oedema including genitalia; nervous signs might appear after the parasite has gone through the blood-brain barrier, resulting in ineluctable death [4, 8–10]. On the contrary, cattle and buffaloes usually develop a chronic form, with depression and reluctance to work, although they can also develop acute forms when the disease is firstly imported to a new area [11]. Among wild species, rhinoceros, deer, wild pig, and Asian elephant may be infected as well and sometimes get severely affected [12, 13]. Consequently, surra's economic impact is high especially in horses. It induces important costs related to mortality, treatment, and prophylaxis as well as limitation of animal movements for reproduction, sales, and touristic or sporting events.

*Trypanosoma evansi* is the main pathogenic trypanosome of livestock in South East Asia (SEA). Despite the important threat it represents, very few governmental veterinary authorities have implemented national control plans to prevent its introduction or to lower its impact. The low specificity of clinical signs induced by *T. evansi* infection, the low sensitivity of the parasitological diagnostic tools, and the lack of reporting by owners or private veterinarians to local veterinary services can explain why veterinary authorities' awareness on this parasite is so low. Therefore, knowledge on the prevalence of *T. evansi* infection in its various host species is needed to better understand the relative roles of those different hosts in the epidemiology of *surra*. In recent times, epidemiological surveys of surra have been conducted in Thailand, in cattle, buffaloes and elephants [14, 15, 16]. However, equines have not yet been covered by these studies.

Based on these considerations, in the present paper, we optimized and applied the antibody ELISA *T. evansi* to equids in Thailand. This test is an OIE-recommended diagnostic method [17] that has already been used and validated for the detection of surra in camels, cattle, buffaloes, and elephants [12, 14, 15, 18]. We then conducted a nationwide seroprevalence survey in Thai equids, including a large group of racing horses and a group of military donkeys and mules working at the border with Myanmar. During the time of the survey, several surra outbreaks were reported to our laboratory. This allowed us to perform treatment evaluations and to develop advice for control measures.

## 2. Materials and Methods

### 2.1. Sample Collection

**Figure 1:**
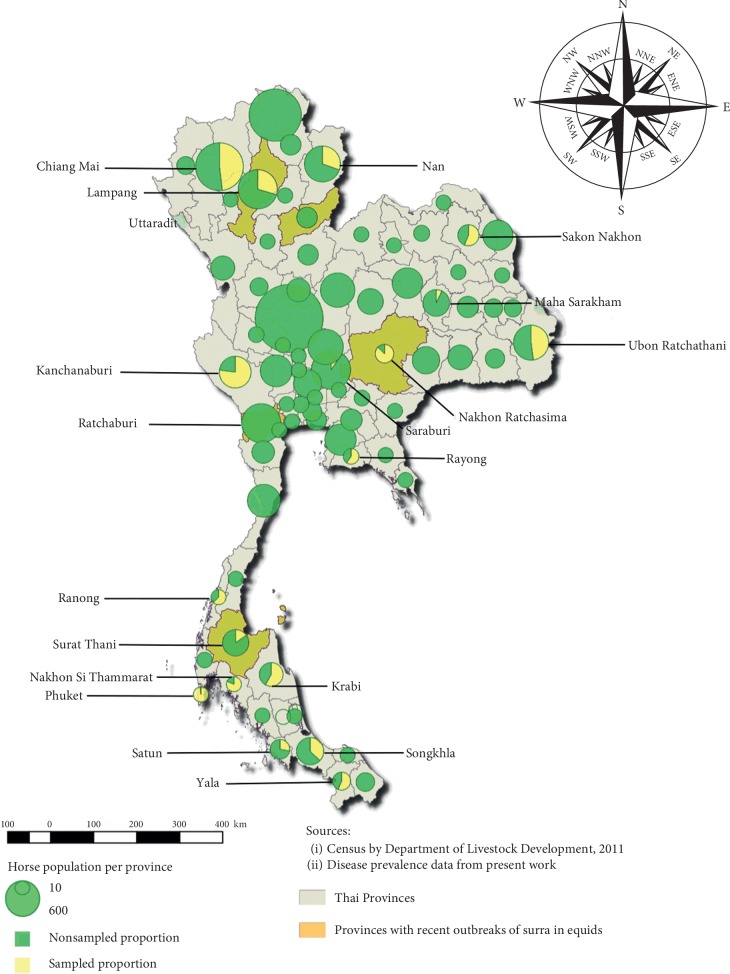
Geographical distribution of horse population and sampled proportion per province in Thailand.

The total horse population in Thailand amounts to 6,503 heads as reported by the Information and Statistics group, Information Technology Centre, Department of Livestock Development, in 2010, at the beginning of the study; they are distributed over the country, as illustrated in Figure 1. But this number is likely to largely underestimate the truth; the actual number would probably be closer to 20,000 heads, as mentioned by professionals working in the horse sector [16]. This population consists of two subgroups, whose raising practices greatly differ. High-value imported breed horses are mainly used for racing or riding and are raised in farms where strict sanitary prevention measures are applied. Local breed horses, whose conformation varies largely depending on the location, usually serve for traditional ceremonies and transportation; they are kept in more extensive conditions. A population of mules and donkeys is also maintained by the army, especially for the surveillance of some remote areas along the border with Myanmar; they are bred with horses in peculiar conditions, and most of them are maintained under permanent chemical prophylaxis due to the high-risk exposure to surra.

Besides random sampling, we also had opportunities to collect samples in suspected or confirmed outbreaks of surra, thanks to a disease surveillance ensured together with the Veterinary Services, Department of Livestock Development (DLD), Thailand. Thus, samples collected during this study belong to two categories:

- Serological survey: samples were randomly collected in farms or in individuals without any current suspicion of surra; among them are 2 subtypes:
  (A1) Racing horses: they are imported breed horses; they were sampled in 2010 by our University team, at the race course in Bangkok. They mainly originated from Nakhon Ratchasima, Saraburi, and Maha Sarakham Provinces, in Central Thailand, where most of the racing horse farms are located. These animals are raised under peculiar care, and they are subjected to continuous veterinary supervision and were apparently healthy on the day of the sampling since they have been selected by their owners to participate in the races. They belong to the HHP category (high-health, high-performance horses), as defined by the OIE.
  (A2) Riding horses: those horses of local or imported breed were sampled between 2010 and 2012 in 17 provinces from Northern, Southern, and Central Thailand by University team, government departments, or local veterinary offices in the framework of regular health check or studies targeting equine infectious anaemia.
- Outbreak or suspect herds: samples were collected by private veterinarians or University teams, between 2010 and 2015, in farms that had a known risk of surra or farms where surra outbreaks were ongoing.
  (B1) Confirmed or suspected surra outbreaks: samples were collected from farms exhibiting confirmed cases or clinical suspicions of surra; most animals on the farm were sampled, treated, and followed-up;
  (B2) Exposed animals: these are equines belonging to groups of working or breeding animals in military camps including mules and donkeys, known as regularly infected and placed under permanent surra chemoprophylaxis.

In all cases, blood was collected from the jugular vein in plain and EDTA tubes; plain tubes were allowed to clot in the shade for 2–4 hours and transferred in an icebox until serum was separated, aliquoted, and stored at −20°C for serological tests. Those samples were used both for standardization of the ELISA test (positive or negative reference samples) and for the epidemiological survey on surra. In addition, to complete their characterisation, these samples were submitted to other classical diagnosis methods recommended by the OIE: parasitological, molecular, and serological tests.

### 2.2. Parasitological, Molecular, and Serological Tests

Blood from the EDTA tubes was centrifuged at 12,000 rpm within less than 3 hours after collection in microhematocrit tubes (Hirschmann® Laborgeräte, Eberstadt, Allemagne) for direct microscopic observation of the buffy coat and measurement of packed cell volume (PCV), according to the Hematocrit Centrifuge Technique (HCT) [19].

Buffy coats were collected from the microhematocrit tubes, transferred into microtubes, and prepared using Chelex® for PCR analyses with Tepan primers [20], according to protocols previously published [21].

The card agglutination test for trypanosomosis/*T. evansi* (CATT/*T. evansi*®, Institute of Tropical Medicine «Prince Leopold», Laboratory of Serology, Nationalestraat 155, B-2000 Antwerp, Belgium) is a direct agglutination test detecting the presence of circulating anti-trypanosome antibodies, mainly immunoglobulin type M [17]. It was performed according to manufacturer's instructions [22] using a serum dilution of 1 : 4. Semiquantitative results were expressed as follows: negative when no agglutination (−) or doubtful agglutination (±) was observed, and positive when weak, medium, or strong agglutination was observed (+, ++, and +++).

### 2.3. ELISA *T. evansi* Optimisation

#### 2.3.1. Protocol

The indirect ELISA *T. evansi*, as described by the OIE [17], has been used in several host species and was already validated for camels, cattle, buffaloes, and elephants in Thailand [12, 14, 15, 18]. The test used in this study was derived from previously published protocols with slight modifications and adaptation to horse samples. In brief, Microtest 96-well PolySorp Nunc® (Nunc, Roskilde, Denmark) were coated with 100 *μ*l/well of locally prepared soluble antigens from whole cell lysate of *T. evansi*, at a protein concentration of 10 *μ*g/ml in carbonate buffer (0.05 M, pH 9.6) and kept for 2 hours at 37°C. The plates were then emptied and blocked with 150 *μ*l/well of blocking buffer (BB) (Phosphate Saline Buffer (PBS) with 5% skimmed milk powder (*Régilait, Saint Martin Belle Roche, France*) with permanent shaking (300 rpm) for 45 min at 37°C. The BB was discarded. Sera diluted 1 : 100 in BB were transferred in duplicate to the ELISA plates. After incubating for 30 minutes at 37°C with permanent shaking (300 rpm), plates were washed seven times with washing buffer (WB) (PBS-0.1% Tween®20 (Sigma-Aldrich)). Then, 100 *μ*l of peroxidase conjugated anti-horse IgG (Sigma-Aldrich A 6917), diluted 1 : 5,000 in BB, was added, and the plates were incubated for 30 min at 37°C with permanent shaking (300 rpm). After washing 5 times with WB, 100 *μ*l of the complex substrate/chromogen 3,3′,5,5′-tetramethylbenzidine (TMB) (k blue® TMB substrate, Neogen Europe Ltd., Scotland) was added. The plates were incubated, without shaking, in a dark room at room temperature for 30 min. Optical density (OD) was measured at 620 nm in an ELISA reader (BIO-RAD iMark®, CA, USA). The mean OD of the two wells (duplicate) for one serum sample was used to express the result of the test.

#### 2.3.2. Selection of Negative and Positive Controls and Expression of Test Results

A pool of reference serum samples from presumed noninfected horses was constituted amongst the local breed horses or imported breed riding horses (Group A2 as defined above). They were defined as samples that were negative to CATT/*T. evansi*, PCR, and parasite direct examination (blood smear and/or Woo technique) [19–22], that originated from farms where no active infection was suspected or detected (PCR or direct examination) and where no recent prophylactic trypanocide treatments had been given. Among the samples meeting those criteria, three serum samples representative of the mean OD (±10%) of the pool of reference serum samples from noninfected horses were selected to constitute a set of 3 negative controls (NC) (medium, high, and low OD).

A pool of reference serum samples from infected animals was selected among the horses in Group B; they were defined as samples from horses undergoing active infection (detected by the direct parasitological method or PCR) at the date of sampling or less than 1.5 months before. Since it has been widely admitted that anti-*T. evansi* IgG can be found in the blood flow more than 2 months postinfection [23, 24], we were guaranteed that those horse samples would still be seropositive. Three samples, representative of the mean OD (±10%) of the pool of reference serum samples from infected horses, were selected as a set of 3 positive controls (PC) (medium, high, and low OD).

ELISAs were performed again, in duplicate for all samples, with the 3 positive controls and the 3 negative controls (selected as indicated above) on each plate. The blank OD value was systematically subtracted from the average OD of each sample, and the results were expressed as a relative percentage of positivity (RPP) as previously described [18] and as follows:

(1) $$\begin{array}{c} \text{RPP sample }=\;\frac{\text{mean OD sample}-\text{mean OD of the }3\text{ NC}}{\text{mean OD of the }3\text{ PC}-\text{mean OD of the }3\text{ NC}}. \end{array}$$

#### 2.3.3. Determination of the Cutoff Line (COL)

To define the optimal COL, we used the indirect ELISA results of all negative and positive reference samples, to perform a two-graph receiver operating characteristics analysis (TG-ROC, [25] with the R package “DiagnosisMed.” The misclassification cost term method was used. The cost ratio of false negative over false positive results was set at 0.5 to favour the test specificity. We considered an *a priori* prevalence of 10%, similar to the values previously observed in buffaloes and dairy cattle in Thailand. Resulting sensitivity (Se) and specificity (Sp) were computed with 95% confidence intervals based on parametric curves simulated with a neural network.

### 2.4. Seroprevalence Survey

Samples collected for the serological survey (Group A), including racing horses (Group A1) and riding horses (Group A2), were tested with ELISA *T. evansi*. The results were expressed in RPP and the COL applied to define positive samples. Apparent seroprevalence rates were directly inferred from indirect ELISA results to evaluate *T. evansi* circulation in each region surveyed.

### 2.5. Outbreaks or Suspect Herds

In farms undergoing surra outbreaks (Group B1), infection rate, mortality rate, and mean ELISA RPP value were calculated. When possible, Nzi and Vavoua traps were placed by University teams nearby animal pens and collected twice a day to identify potential *T. evansi* vectors and evaluate their abundance. In addition, the percentages of recovery after the different treatments were compared to conclude on the most effective treatment based on the epidemiological situation.

We also reported the results of diagnosis carried out in mules, donkeys, and horses from military camps in which recent history of surra was documented, and chemoprophylaxis was applied (Group B2).

## 3. Results

### 3.1. ELISA *T. evansi* Optimisation

For ELISA optimisation, a pool of 1,382 serum samples from presumed noninfected horses and a pool of 96 serum samples from infected horses were identified in agreement with the definitions presented under Section 2.3.2. Control samples were then selected as follows (results expressed in optical density):

  Negative control samples (mean OD 0.057): NC1 (low), 0.053; NC2 (medium), 0.058; and NC3 (high), 0.060
  Positive control samples (mean 1.204): PC1 (low), 0.791; PC2 (medium), 1.109; and PC3 (high), 1.411.

ELISA was then performed again, and the results expressed in RPP vis-à-vis the three positive (PC) and the three negative control samples (NC).

**Figure 2:**
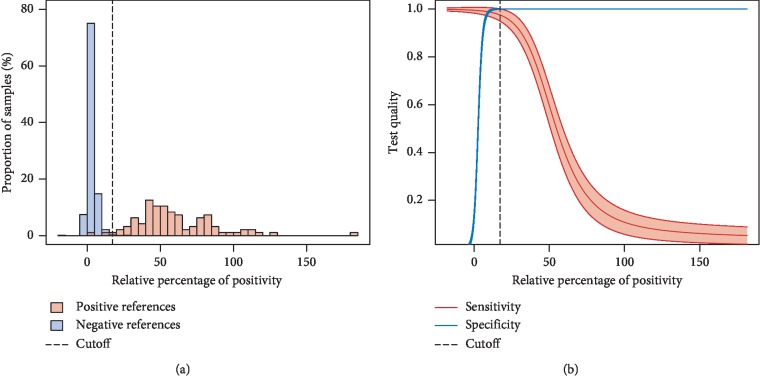
(a) Distribution of ELISA's relative percentage of positivity (RPP) values for horse reference samples and (b) TG-ROC curves: estimates of sensitivity and specificity based on the RPP cutoff. The optimal cutoff according to the misclassification cost term (MCT) criteria is indicated.

Mean ELISA RPP of 1,382 reference serum samples from presumed noninfected horses was 3.0% ±0.15, while that of 96 reference serum samples from infected animals was 59.6% ± 5.50. RPP values of presumed noninfected and infected references samples slightly overlapped (Figure 2(a)). According to the TG-ROC analysis, the optimal cutoff line (COL) was 17.3%, and resulting sensitivity and specificity were estimated to be 97.5% (95% CI: 94.9–100%) and 100% (95% CI: 99.9–100%) (Figure 2(b)).

### 3.2. Seroprevalence Survey

For the serological survey, a total of 1,961 individuals were collected over 18 provinces. The number of horses sampled by the province ranged from 7 to 435, for a mean of 108.9 (SD = 119.25) (Figure 1). Horse age ranged from 1 month to 28 years for a mean of 6.7 years (SD = 4.5). Females represented 56.2% of the animals. The overall seroprevalence observed in ELISA *T. evansi* in this horse survey was 0.36% (7/1,961) (95% CI: 0.1–0.7%).

Only one animal, from Pa Phayom District, Phatthalung Province (South of Thailand), exhibiting clinical signs, was found infected by parasitological examination and was strongly seropositive in ELISA *T. evansi* with a RPP of 129%. On the day of sampling, it was treated with DA 7 mg/kg and was further followed-up by local practitioners.

Beside this case, all parasitological examinations were negative and only another six animals were found seropositive with ELISA *T. evansi*: three in Kanchanaburi (RPPs: 20, 21, and 27%), two in Chiang Mai (RPPs: 26 and 36%), and one in Lampang (RPP: 17.42%). The mean ELISA RPP of these animals was low (25%), especially compared to the infected animal mentioned above (RPP: 129%). The seropositive animal found in Lampang was reported as presenting surra clinical signs two months before the sampling and had received trypanocide treatment (Antrycide®) at that time; its very weak seropositivity, just above the COL, could be interpreted as postinfection/treatment-persistent antibodies, however, at a very low level. The two seropositive horses from Chiang Mai and the three from Kanchanaburi belong to military camps; however, they were not linked with exposed animals (mule, donkeys, or horses working at the border with Myanmar) since they are riding horses. Their seropositivity is weak to medium (20–36%), indicating that they may have been exposed to the parasite in the recent past; however, we could not get more information or follow-up.

Amongst the 282 samples from racing horses brought to Bangkok for racing, none exhibited any seropositivity. Results obtained with the CATT *T. evansi* were 9.2% (26/282) of seropositive samples in racing horses (Group A1) and 5.6% (95/1679) in the rest of the horse population (Group A2).

### 3.3. Outbreaks

Sufficient data were collected in two of the five outbreaks reported to our laboratory to be described here. The first occurred in April 2011, in a mixed farm, in Surat Thani Province. In this farm, horses were pasturing freely together with cattle and goats. Private veterinarians had been called by the owner after several horses had exhibited clinical signs such as fever, anorexia, weight loss, anaemia, bloody urine, and dependent oedema, in early 2011. At the time of sampling (21 April 2011), six horses were found infected by *T. evansi* through the Woo technique and PCR and were seropositive in ELISA *T. evansi*, with a mean RPP as high as 65%; five of them were also positive by CATT. Detailed information is reported in Table 1. All positive horses were treated on several occasions with diminazene aceturate—Berenil® (DA) at dosages varying from 3.5 to 7 mg/kg, or melarsomine hydrochloride–Cymerlarsan® (MH) at 0.25 mg/kg in May and June 2011. Recovery from both treatments was always transient, and relapses were irregularly observed, until 11 of the 12 horses were shown to be infected by the Woo technique on 17^th^ August 2011. The situation evolved toward 92% morbidity and mortality and 100% lethality. Indeed, amongst the 12 horses in the farm, 11 were infected and died. The horse that was still alive by the end of the follow-up was the youngest one, which was never found to be infected nor expressed clinical signs; it stayed seronegative throughout the follow-up. The origin of the outbreak was presumed to be the introduction of one cattle several months earlier, which exhibited clinical signs of lethargy and anorexia some weeks after introduction, but was not identified as infected by *T. evansi* at that time. Insect traps that were installed early in the course of the outbreak (June 2011) for a few days allowed the identification of *Tabanus megalops* and *Stomoxys* spp.*; S. calcitrans* was in a greater proportion than *S. indicus*; only 15 to 20 potential vectors (tabanids or *Stomoxys* spp.) were caught per Nzi trap per day in June 2011.

The second outbreak occurred in August 2011, in Ratchaburi Province, in a farm where 17 local horses were pasturing together with zebu cattle. Most of the horses (13/17) exhibited clinical signs including fever, anorexia, lack of energy, and oedema although no nervous sign was spotted yet. Two of them died early. When the other 15 horses were sampled, 40% were found positive in parasitology (HCT), 73% by PCR, and 80% (12/15) in ELISA *T. evansi*, with a mean RPP of 54%; 53% were positive by CATT. Detailed information is reported in Table 1. After isolation of four horses from the cattle reservoir, thanks to fly-proof stables that the owner agreed to build (stables were completely covered by a mosquito net), these animals were treated successively with 2 injections of DA at 3.5 mg/kg and 7 mg/kg at a one-week interval. Relapses occurred after a short remission, leading to appearance of nervous signs in two horses and death of one of them. MH at 0.5 mg/kg was administered to the three remaining horses allowing remission in all of them. However, the horse that had previously shown nervous signs relapsed early, and despite a treatment with Quinapyramine sulphate and chloride (Triquin®), it could not be cured and finally had to be euthanized. The other 2 horses fully recovered and were considered as cured, giving a 67% (2/3) treatment efficacy to MH treatments at 0.5 mg/kg. The overall mortality rate of this outbreak in horses was 47% (8/17); all surviving horses were treated preventively with quinapyramine sulphate and chloride, Triquin® (QSC) which proved to be efficient as no infection developed in horses later on. Finally, amongst 17 horses, 15 had been infected, nine died, six recovered, and two remained uninfected. It can be noted that all horses that proved to be infected (13/15) had been detected using ELISA as early as eight days after the outbreak was identified.

### 3.4. Early Clinical Suspicions

Three surra cases were suspected by veterinarians early in the course of the infection; they were reported in Lampang, Ratchaburi, and Uttaradith farms, respectively, in August, September, and October 2015. In each case, only one animal was found infected and was positive by using the PCR technique (negative by HCT), ELISA *T. evansi* and CATT *T. evansi,* resulting in, respectively, 20% (1/5), 17% (1/6), and 11% (1/9) of infected and seropositive horses. The three infected horses showed RPP of 55%, 104%, and 43%. Quinapyramine treatments were administered early, and the animals fully recovered.

### 3.5. Survey in Mules, Donkey, and Working Horses of the Military Camp

A total of 242 horse, 41 donkey, and 459 mule samples were collected from working, training, or mixed breeding groups at the military camp of Chiang Mai, in December 2011. In the military camp, equines were gathered in 12 groups of 13 up to 130 individuals pasturing together. Among those animals, 7.8% (19/242) of the horses, 31.7% (13/41) of the donkeys, and 1% (5/459) of the mules were seropositive by ELISA *T. evansi*, as reported in Table 2. However, most seropositive animals belonged to the same group (Group 11), constituted of 13 donkeys and 19 horses that were all seropositive and under constant chemoprophylaxis using quinapyramine. This group was obviously a reservoir of the parasite. Other seropositive samples were five mules belonging to two other working groups (Groups 1 and 2). Finally, the seroprevalence pattern was quite contrasted since it was nil in Groups 3, 4, 5, 6, 7, 8, 9, 10, and 12, very low in the mule Group 1 (3.1%) and Group 2 (0.08%), but as high as 100% in the mixed reproduction Group 11.

## 4. Discussion

### 4.1. Optimisation

The indirect ELISA *T. evansi* was optimized and used in a protocol derived from the original description made in the Terrestrial Manual of the OIE, Chapter 2.1.17 (OIE 2012). During such test optimisation, the selection criteria for negative and positive reference populations may clearly impact COL, sensitivity, and specificity obtained. Since negative reference samples were obtained from animals negative to parasitology, PCR, and CATT, in farms which had no known exposure or history of surra, false-negative samples were not likely to be included. Indeed, surra being an acute disease in horses, farmers, and veterinarians would not have missed clinical signs. Similarly, positive reference samples were being obtained from animals with positive direct examination or PCR, or from animals which recently proved to be infected, and false-positive samples were not likely to be included neither. Reliable reference samples favour the development of a test presenting good positive and negative predictive values. Indeed, amongst infected animals, 97.9% (94/96) were seropositive by ELISA (versus 81.2% (78/96) using the CATT *T. evansi*); this result is consistent with the sensitivity of the ELISA *T. evansi* evaluated under the TG-ROC analysis, which is 97.5%. On the contrary, the fact that none of the 282 racing horses exhibited any seropositivity in ELISA (versus 9.2% (26/282) using the CATT *T. evansi*) confirms its high specificity; this result is consistent with the specificity of 100% of the ELISA *T. evansi* evaluated under the TG-ROC analysis.

These results highlight the usefulness of the ELISA *T. evansi* for screening surra infections in horses. Nevertheless, delayed antibody response of horses to *T. evansi* infection has already been reported, with specific IgG being detected in the blood only from day 10 to 20 postinfection [24, 26]. Consequently, recently infected animals might not be detected by the test, leading to a lack of sensitivity in the very early stage of the infection. However, such animals are easily detected as positive when using parasitological tests or PCR.

### 4.2. Seroprevalence Survey

Result of the seroprevalence survey, 0.36% seropositive animals, suggests a very low, if any, circulation of the parasite in horses. Seropositive animals detected in this survey have a very low probability to be false positive since the test was shown to be highly specific (Sp = 100%). The seropositive animals that have been infected are most probably horses and have cured early some weeks or months before sampling. Such low seroprevalence was expected since *T. evansi* infection in horses causes strong clinical signs, most often followed by death, if animals are not diagnosed and treated early enough. This high pathogenicity of *T. evansi* in horses was observed in outbreaks that were investigated, where lethality reached very high levels (100% in one case), whereas it was nil in nearby infected cattle. Thus, there is a very low probability that chronically infected individuals or healthy carriers remaining in the horse population.

In other epidemiological circumstances, such as those described in India, horses have been able to develop an immune response that allows them to survive despite the infection, either as chronically infected individuals or even healthy carriers [27]. These surviving animals may become a source of infection for others equines, as the parasite could reemerge later as a result of a stress. Such asymptomatic portage has never been reported in Thailand so far; horses under permanent chemoprophylaxis in Chiang Mai military camp (see below § 4.5) are the only seropositive and apparently healthy equines that were observed.

### 4.3. Comparison of CATT and ELISA Results

Among racing horse, 0% of the samples was ELISA positive, whereas 9.2% (26/282) was CATT positive. Those “high-health, high-performance horses” (HHP), as defined by the OIE, are raised without contact with other host species and were considered by their owners healthy enough to participate to racing events. In addition, they all exhibited high PCV and proved to be negative by parasitological and molecular tests. We can therefore assume that they are not nor have recently been infected by *T. evansi*; consequently, these CATT positive samples in racing horses are most probably false-positive results. If CATT was to be used for screening purposes, it might wrongly disqualify a significant number of animals from racing. Such lack of specificity of the CATT has already been observed in horses and other host species [28–31] and is rather common in rapid agglutination tests [32, 33].

Among the 96 samples from infected animals, ELISA detected 94, exhibiting a sensitivity of 97.9%, while the CATT detected only 78, exhibiting a sensitivity of 81.2%. This lack of sensitivity is consistent with previous observations made in various hosts [29, 34–38]. It is mainly explained by the fact that the IgM level in the serum is highly fluctuating due to the formation of immune complexes which are actively phagocytised so that irregular positivity is observed with CATT during the course of the infection [26]. Nevertheless, CATT is a point-of-care diagnosis method, easy to use, and can be used in first intention in remote areas where immediate results are needed or in confirmed outbreaks, in order to identify infected animals for treatment decision. Moreover, the positive predictive value of the CATT is generally considered as high, especially in horses [39], and at the early stage of the infection, so it is of value to use the CATT in an outbreak situation, in order to detect infected animals as early as possible.

ELISA's specificity and sensitivity make it a more reliable test for screening purposes; indeed, the 100% specificity of the test will properly qualify noninfected animals, and its high sensitivity will ascertain an animal's infected/noninfected status, if the test is repeated twice at a 3-4-week interval, allowing recently infected animals to build up immune response.

### 4.4. Outbreak Follow-Up and Treatment Evaluation

In the two outbreaks described, the high infection rates demonstrated the very quick and effective mechanical transmission between horses and cattle. Indeed, cattle and buffaloes seem to be the main source of parasites for the initial contamination of horse herds, as regularly observed in Thailand [14, 15, 40, 41]. Thus, equines should have pastures separated from bovine ones. Should horses and bovines be raised in close proximity, precautions are to be taken: chemoprophylaxis or fly-proof stables are the two options to consider for horses.

*Stomoxys* spp. and *Tabanus megalops* were trapped and identified on Surat Thani outbreak site; these species have been recognized as vectors of *T. evansi* [11]. The apparent insect density per trap per day obtained in our study (15 to 20 *Stomoxys* spp. and *Tabanus* spp.) was compatible to those obtained in other studies on the Nzi trap efficiency [42, 43] and allowed for a very effective mechanical transmission.

Regarding treatment, diminazene aceturate is the most employed trypanocide drug in Thailand; a first injection at a dose of 3.5 mg/kg is usually recognized to gently reduce the parasitaemia in heavily infested animals, whereas a second injection at 7 mg/kg a few days later is supposed to clear off parasites. From our experience, in more than half of the cases, DA proved to be ineffective in treating *T. evansi* infected horses, even at the highest dosage. Either no improvement of clinical signs or early relapse was observed, after a short remission period. In Surat Thani outbreak, reoccurrence of infection after treatment could be attributed to parasite resistance to DA or to reinfection from the cattle reservoir that had not been treated nor isolated from horses [44]; however, since the horses found infected after treatment were the same horses as before treatment, the hypothesis of a parasite circulation has a lower probability than relapse due to inefficient treatment. In Ratchaburi, since horses were placed under mosquito net protection, the hypothesis of reinfection could be eliminated; consequently, we could conclude on parasite resistance to DA, as previously reported [40, 41, 45].

Melarsomine hydrochloride is a more recently commercialised trypanocide drug that is unfortunately unavailable in Thailand, except for research purposes. It has been used in cattle and buffalo elsewhere. Relapses have been observed after treatment at 0.5 mg/kg in those species but not in horses, and it was advised to administer a dose of 0.75 mg/kg to prevent this phenomenon in buffaloes [46–48]. In our case, the subcutaneous injection of MH at 0.5 mg/kg to three horses under fly-proof conditions in Ratchaburi outbreak allowed to conclude on the efficacy of this treatment in horses as long as they are not presenting nervous signs, i.e., as long as the parasites have not gone through the blood-brain barrier; after what there is no possibility of successful treatment, even using quinapyramine [49]. This last drug is largely used in Thailand for preventive purposes; quinapyramine sulphate and chloride are usually administered every four months, and no resistance phenomenon has been reported so far.

### 4.5. Follow-Up of Mules, Donkey, and Horses from the Military Camp

Very different pictures were observed when comparing seroprevalence in the different groups of equines from Chiang Mai military camp. Indeed, it is suspected that working and breeding mules, donkeys, and horses that often travel along the Myanmar border are repeatedly exposed to *T. evansi* infection. However, large groups of working mules were not severely infected (seroprevalence <4% in eight of the nine mule groups), while the group of breeding donkeys and horses exhibited a seroprevalence of 100%. This group is placed under permanent chemoprophylaxis using quinapyramine, which seems to be the only alternative to keep the horses alive under such epidemiological situations. It is suspected that the donkeys may constitute an active reservoir of the parasite from which it can be easily transmitted by mechanical vectors, but also possibly during sexual intercourse. Determining the exact effect of the quinapyramine under such circumstances is still questionable since we observe presence of antibodies despite regular administration of the drug to all animals. It could be hypothesised that parasite chemoresistance to the drug allows occasional circulation of the parasites that induces build-up of an immune response, which also helps the animal to contain the parasites and avoid the appearance of clinical signs.

## 5. Conclusion

The ELISA *T. evansi* used in this study presents very high specificity (100%) and sensitivity (97.9%), which qualifies it as a sensitive and specific diagnosis method for detection of surra infection in equines. Using horse immunoglobulin conjugate provided satisfying results in both horses, donkeys, and mules although the test was not standardized for the latter. Conversely, the CATT *T. evansi* presented a significant rate of false-positive results which is not suitable to provide a reliable immune status in noninfected horses. Based on a the very high specificity of the test, ELISA *T. evansi* can be proposed for screening horses prior to international animal movements, with a very low risk of unjustified rejections. Also, based on its high sensitivity, ELISA *T. evansi* can be proposed on its own as a secure mean of detection of *T. evansi* infection, providing it is run twice at 3-4-week interval during a quarantine (according to the dynamic of the immune response).

Results of this study are consistent with prior knowledge stating that surra is almost always fatal to horses and that horse infections originate from bovines, or possibly from donkeys and/or mules. Due to the obvious clinical evolution of any horse infection by *T. evansi*, serological survey is of little help for surra management in horses. However, ELISA can be useful for posttreatment follow-ups, in order to confirm their efficacy. CATT *T. evansi* remains useful to detect early infections and thus for treatment decision in recent outbreaks and can also be used for posttreatment follow-ups.

The following guidelines for the management of surra outbreaks could be useful to the local veterinary offices. It is particularly advisable to avoid horses pasturing with cattle (or buffaloes) since they act as reservoir of the parasites, while the horses act as sentinel animals due to their very high receptivity and susceptibility. Donkeys may also be an efficient reservoir, so they should be cured or placed under chemoprophylaxis protection (quinapyramine) if they are to pasture or mate with horses. Also, identification and isolation of infected and treated animals are key points to be able to differentiate relapse from reinfection and to protect uninfected animals; treatment of all animals present on the farm on the same day could be an alternative to this isolation measure. Diminazene aceturate can still be used in cattle and buffaloes at 7 mg/kg but not at a lower dose; this drug should no longer be used in horses as the parasite can resist to higher doses, and the horses are highly sensitive to the toxic effects of DA. However, serial low-dose treatment protocols may be attempted if the objective is to enhance the immune status of the equids against surra. Melarsomine hydrochloride should be used in horses in first intention at 0.5 mg/kg, and quinapyramine at 5 mg/kg should be used to protect the exposed animals only. In all cases, treatment is useless once neurological signs have appeared since a fatal outcome is always observed.

## Figures and Tables

**Table 1:** Summary of data from 2 outbreaks of surra in horses in Thailand.

Location		Surat Thani	Ratchaburi
Starting date		April 2011	August 2011

Total number of horses in the farm	12	17	

Positive rates (*n*/*N*) at *t*0	Stained blood smear	27% (3/11)	ND
	HCT	36% (4/11)	40% (6/15)
	CATT	45% (5/11)	53% (8/15)
	ELISA	55% (6/11)	80% (12/15)
	PCR	55% (6/11)	73% (11/15)

Positive rates at *t*0 + # months	CATT	*t*0 + 4 months, 67% (8/12)	*t*0 + 1 month, 64% (7/11)
	ELISA	92% (11/12)	81% (9/11)
	HCT	92% (11/12)	36% (4/11)

Percentage of horses exhibiting clinical signs (*n*/*N*)		92% (11/12)	87% (13/15)

Percentage of horses exhibiting nervous signs (*n*/*N*)		0% (0/12)	13% (2/15)

Treatments applied	DA	3.5 mg/kg and 7 mg/kg	3.5 mg/kg and 7 mg/kg
	MH	0.25 mg/kg to 4 horses	0.5 mg/kg + fly-proof stables for 4 horses

Morbidity rate (*n*/*N*)		92% (11/12)	88% (15/17)

Mortality rate (*n*/*N*)		92% (11/12)	53% (9/17)

Lethality rate (*n*/*N*)		100% (11/11)	60% (9/15)

Recovery rate (*n*/*N*)		0% (0/11)	40% (6/15)

Remarks		Relapse of parasites in blood and clinical signs observed in most of the horses, 2-3 weeks after each treatment	1 out of 4 horses exhibited nervous signs before MH treatment; QSC 5 mg/kg used as curative and preventive treatment

*n* = number of animals positive; *N* = number of animals tested or observed; ND = not done.

**Table 2:** Results of ELISA *T. evansi* in 12 groups of mules, donkeys, and horses occasionally exposed to surra infection (# positive/# tested (seroprevalence %)) in Chang Mai.

Group number	Mules	Donkeys	Horses	Equines
Group 1	4/130 (3.1%)	—	—	4/130 (3.1%)
Group 2	1/123 (0.08%)	—	—	1/123 (0.08%)
Group 3	0/25 (0%)	—	—	0/25 (0%)
Group 4	—	—	0/13 (0%)	0/13 (0%)
Group 5	—	—	0/66 (0%)	0/66 (0%)
Group 6	0/49 (0%)	—	0/1 (0%)	0/50 (0%)
Group 7	—	—	0/79 (0%)	0/79 (0%)
Group 8	0/27 (0%)	—	0/21 (0%)	0/48 (0%)
Group 9	0/40 (0%)	—	—	0/40 (0%)
Group 10	0/7 (0%)	0/2 (0%)	0/31 (0%)	0/40 (0%)
Group 11	—	13/13 (100%)	19/19 (100%)	32/32 (100%)
Group 12	0/58 (0%)	0/26 (0%)	0/12 (0%)	0/96 (0%)
Global	5/459 (1%)	13/41 (31.7%)	19/242 (7.8%)	37/7412 (5%)

## Data Availability

Data supporting the conclusions of this paper are not available online but can be asked directly to authors.

## Additional Points

*Highlights*. (i) Optimisation of the ELISA *T. evansi* in equids was implemented, and protocol was described. (ii) Under this protocol, ELISA *T. evansi* in equids presented 97.5% sensitivity and 100% specificity. (iii) Low to nil circulation of *T. evansi* in horses in Thailand (*P*=0.36%), due to the high lethality rate. (iv) Reliability of the ELISA test to qualify noninfected equids was demonstrated, notably in racing horses. (v) Parasites' resistance to diminazene aceturate was confirmed. (vi) Melarsomine hydrochloride is efficient to treat horses in the absence of nervous infection.
